# How maternal adversity impacts offspring

**DOI:** 10.7554/eLife.51206

**Published:** 2019-09-25

**Authors:** Zaneta M Thayer, Chlöe A Sweetman

**Affiliations:** 1 Department of AnthropologyDartmouth CollegeHanoverUnited States; 2 Ecology, Evolution, Environment and Society ProgramDartmouth CollegeHanoverUnited States

**Keywords:** early adversity, intergenerational effects, developmental constraints, maternal effects, survival, <i>P. cynocephalus</i>

## Abstract

Adversities experienced by female baboons early in life can affect the survival of their offspring years later.

**Related research article** Zipple M, Archie EA, Tung J, Altmann J, Alberts SC. 2019. Intergenerational effects of early adversity on survival in wild baboon. *eLife* **8**:e47433. doi: 10.7554/eLife.47433

Surviving to adulthood is not an easy task, particularly for animals that live in the wild. For instance, among baboons born in and around Ambolesi National Park in Kenya, only 50% of females and 44% of males will make it to adulthood (Alberts, 2019). In order to survive, individuals must first be born healthy, have access to sufficient nutritional resources, and avoid predation. Baboons and other species, including humans, rely on the extensive care provided by parents to protect them from these challenges, and to teach them the skills they need to thrive in complex social and ecological environments. Given the important role that parents, particularly mothers, play in the growth, development, and survival of their offspring, what happens when mothers have themselves experienced significant challenges in their own early life?

Now, in eLife, Susan Alberts and co-workers at institutes in the US and Kenya – including Matthew Zipple (Duke University) as first author – report how adverse experiences in early life among female baboons affects offspring survival (Zipple et al., 2019). They analyzed data collected from wild baboons in and around Ambolesi Park across four decades, and measured various examples of adversity that had previously been associated with reduced survival among female baboons: maternal death; having a low social rank; experiencing high levels of competition for resources; being born in a drought; and having a close-in-age younger sibling (Tung et al., 2016). The data revealed that the challenges faced by the mother were more strongly associated with offspring survival than the offspring’s own experiences of adversity.

One explanation for this could be that offspring have evolved to be sensitive to cues their mother provides about the quality of the environment (Mousseau and Fox, 1998; Figure 1). The potential for these intergenerational effects is even greater in mammals, where pregnancy and breastfeeding allow for maternal biology to influence offspring development through the transfer of hormones. If mothers live in an environment with high adversity, maternal hormones can provide the offspring with ‘predictive’ cues about its future environment and change how the offspring grows and develops (Kuzawa, 2005).

**Figure 1.**
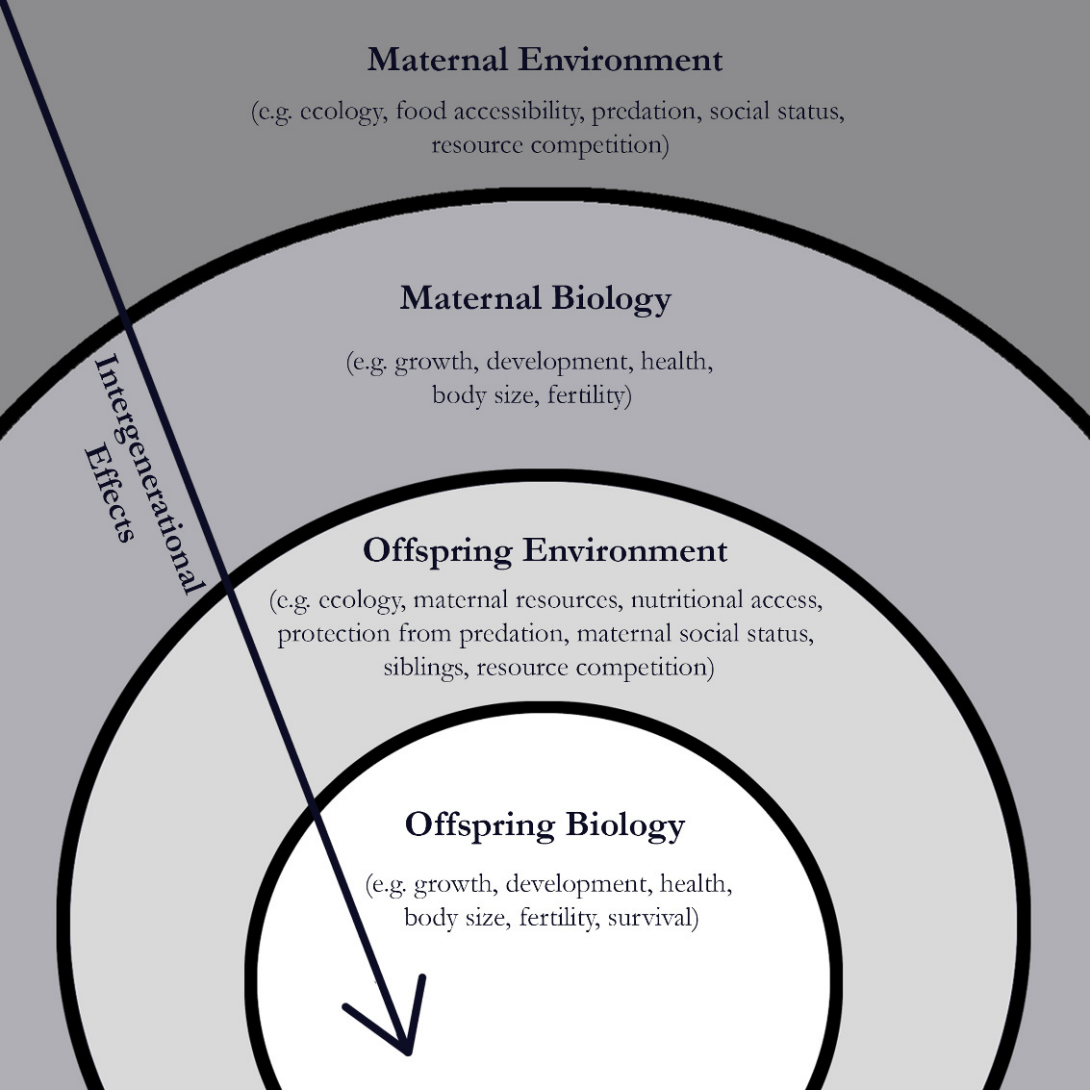
Early adversity experienced by female baboons can have an impact on their offspring. When a baboon experiences adversity in its environment (such as predation or a lack of food), there is an impact on its biology (such as its growth and development). Zipple et al. report that when a female baboon experiences adversity early in her life, there can be an impact on the survival of her offspring. Image credit: Chlöe Sweetman (CC BY 4.0)

Zipple et al. also found that offspring were less likely to reach adulthood if their mother’s own mother had died, or if their mother had a close-in-age younger sibling. This finding, however, is not consistent with the idea that changes to offspring biology are only caused by predictive cues provided by the mother. Instead, it illustrates how diminished access to resources in early life can have a cascading effect on survival that persists across generations (Figure 1). For example, female baboons faced with the loss of their own mother or the quick arrival of a resource-needy sibling could experience greater nutritional stress, which critically limits their growth and development (Gagliano and McCormick, 2007). As a result, when they become mothers these baboons may struggle to provide the resources their own offspring need.

While not investigated by Zipple et al., early adversity could also reduce the quality of maternal care. Early maternal death and the birth of a close-in-age sibling, for example, could result in an individual receiving less care, and not learning how to care for their own offspring. Finally, mothers who experienced early adversity are also more likely to experience early mortality, suggesting that offspring death may be a result of mothers no longer being able to directly protect and provide for their offspring (Zipple et al., 2019).

Work by Alberts on the same population of baboons has revealed that mothers who experienced early adversity were also more likely to be socially isolated from other females in adulthood (Alberts, 2019). As well as reducing their own survival, this social isolation could prevent female baboons from bonding with other mothers, which may influence the health and survival of their offspring. For example, reduced social bonds could result in less grooming of offspring, which could increase parasites, such as ticks. Grooming also affects microbiome diversity among baboons, suggesting that a reduction in communal grooming could lead to immune system or metabolism changes that impact the offspring’s health (Tung et al., 2015).

These findings suggest that the stressful environments experienced by a mother can negatively impact offspring survival. Future work should focus on investigating precisely how adversity early in life affects patterns of maternal care, and to what extent these effects influence the support and care non-mothers provide to offspring.
